# Evaluation of the Effects of Mobile Smart Object to Boost IoT Network Synchronization

**DOI:** 10.3390/s21123957

**Published:** 2021-06-08

**Authors:** Francesco Lamonaca, Domenico Luca Carnì

**Affiliations:** Department of Informatics, Modelling, Electronics and Systems Science, University of Calabria, 87036 Arcavacata, Italy; dlcarni@dimes.unical.it

**Keywords:** synchronization, consensus, mobile object, IoT network synchronization, IoT network topology

## Abstract

This paper deals with the synchronization of Mobile Smart Objects (MSOs). Today, this scenario is becoming typical in Industrial IoT applications due to the plethora of MSOs available as robots, drones and wearables, equipped by sensors making them measurement instruments cooperating in distributed measurement systems. In this context, the synchronization accuracy is directly tied with the accuracy of the performed measurements. In hierarchical synchronization approaches, the presence of an MSO makes the network topology time varying, and this could prevent the synchronization of the whole network. Peer to peer approaches do not need node hierarchy to synchronize but could not converge to a common sense of time. To overcome these challenges, this paper proposes a consensus-based approach for which the convergence to a common sense of time is here demonstrated. The proposal deploys the MSO to bring the common sense of time from an SO to another, establishing new paths among SOs. The new paths are temporary and depend on the MSO’s route. In the paper, the influence of the MSO’s route on the synchronization accuracy *σ* and the time interval to synchronize all the SOs ∆*_TIS_* is investigated, also. The mathematical proof, the simulations and the experimental tests confirm that the MSO can reduce both the values of *σ* and ∆*_TIS_*, because the new connections introduced by the MSO can boost the exchange of information among SOs. Consequently, the criteria to a priori select the route ameliorating *σ* and ∆*_TIS_* values are proposed.

## 1. Introduction

Accurate time synchronization is a key enabler of real-time Industrial IoT applications [1,2,3,4], and numerous applications are available [1,5,6,7,8,9,10]. It permits the coordination in time between sensors and actuators equipping Smart Objects (SOs) [8,9,11] different SOs allowing the modelling of the system, and the design of the control systems [6,12,13,14]. Moreover, the collection of the measurements of a big number of SOs (Big Data) allows data retrieval [15,16,17] if their sampling time is accurately defined.

In recent years, low-power wireless SOs have gained popularity [18]. They interact to form large networks such as Machine to Machine (M2M) networks and Wearable Computing. In these contexts, the major challenge is to guarantee robust communication between elements while keeping the whole network energy efficient. The time synchronization permits them to coordinate communication slot for the SOs [4], implementing a time division multiple access communication protocols, i.e., the SO has to forward messages to its neighbors in only a short wakeup period.

The time synchronization of the measurements requires that all the network SOs share the common sense of time. The synchronization accuracy *σ* among SOs affects the efficacy of the data retrieval, while the time interval required to synchronize all the SOs (∆*_TIS_*), i.e., the time interval starting with the execution of the synchronization algorithm and ending when the delay between any couple of SOs is lower than the admissible delay range, affects the “warm-up” time of the system. In the time interval ∆*_TIS_*, no action should be performed, because the achieved data can refer to different time instants.

These days, time synchronization in IoT wireless network must take into account that several SOs can be Mobile SOs (MSOs). For example, moving robots are used in a warehouse to move goods [19,20] autonomous unmanned aerial vehicles (drones) [21,22] coordinate among themselves to achieve a common task, wearables are used to monitor health parameters [23,24,25], and so on.

The MSO makes the topology of the network varying in time. In this context, promising solutions are the consensus approaches since they are based on the local exchange of messages among SOs [26,27]. In [26], the MSO navigates toward each SO deployed on a straight-line route, bringing the sense of time of the previous visited SO. In [27], a bidimensional deployment of the SOs is considered, and the MSO moves on a straight-line route. The research proposed in [26,27] highlights that the MSO reduces ∆*_TIS_* [28].

The novelties of this paper consist in: (i) proofing the convergence of the proposed synchronization method, (ii) considering that the MSO can move on a bidimensional route (that is a typical scenario), and (iii) detecting the update condition that MSO has to satisfy to promote the SOs’ synchronization, in the case it executes for the first time the synchronization procedure.

Moreover, in the paper, it is proved that the increased number of paths not only reduce ∆*_TIS_* but could increase the synchronization accuracy *σ*. This is conceptually justified by the fact that, during the synchronization, the MSO visit a SO, and it is connected with it and all SOs in its Broadcast Range (BR). The MSO (i) takes part to the network synchronization and (ii) increases the number of connections among the set of SOs in its BR and, consequently, the number of paths among SOs. If the MSO makes higher the number of paths among SOs, faster is the propagation of the common sense of time: the influence of the sense of time of a SO on all the others occurs in a lower time interval. As a consequence, the effect of the connections introduced by the new time-varying paths on *σ* and ∆*_TIS_* is analyzed. In particular, to link the MSO’s route to the improvement of the two synchronization parameters ∆*_TIS_* and *σ*, two statistical indexes are defined: the average of the minimum number of connections in the path between any couple of SOs *μ_con_*, and its variation *σ_con_* versus the time. Based on such approach, the criteria to select more convenient MSO route are given.

The paper is organized as follows: In Section 2, the hardware and software architectures of both SO and MSO and the synchronization procedure are abstracted. In Section 3, the effect of the MSO on the synchronization is analyzed. In Section 4, numerical tests are executed to evaluate the effect of the MSO route on ∆*_TIS_* and *σ* together with *μ_con_* and *σ_con_* to detect the more convenient criteria for the MSO route selection. In Section 5, experimental results are presented to assess the theoretical and numerical results in a real-world scenario. The conclusions follow.

## 2. SO and MSO Architecture and Synchronization Procedure

Concerning the synchronization purpose, both the architectures of the SO and MSO can be related to the node architecture of [29] and can be modeled by three sections: clock, μ-processor, and radio. With respect to the SO, the MSO’s architecture also includes the hardware and software allowing the movement along the route.

The clock is typically composed of a nontunable crystal oscillator [30] driving a counter. The output of this counter is used as a hardware clock. For the *i*-th SO, SO#i, at time *t*, the hardware clock value *τ_i_* is:

(1) $$\tau_{i}(t)=\left\lfloor \alpha_{i}t\right\rfloor +\beta_{i},\;i=1,\ldots ,\;Nc,$$

where ⌊⋅⌋ is the round toward negative infinity operator, *Nc* is the number of SOs in the IoT network, *α_i_* is the frequency, and *β_i_* the offset of the clock.

Since *α_i_* and *β_i_* vary according to the actual realization of the crystal oscillator and the starting condition of the SO#i, respectively, the μ-processor acts as clock-servo, evaluating the correction parameters for frequency ${\hat{\alpha }}_{i}\left(t_{k}\right)$ and offset ${\hat{o}}_{i}\left(t_{k}\right)$, respectively. The μ-processor computes the software time ${\hat{\tau }}_{i}(t)$ according to:

(2) $${\hat{\tau }}_{i}(t)={\hat{\alpha }}_{i}\left(t\right)\tau_{i}\left(t\right)+{\hat{o}}_{i}\left(t\right),$$

The correction parameters ${\hat{\alpha }}_{i}\left(t\right)$, and ${\hat{o}}_{i}\left(t\right)$ are evaluated by each SO on the basis of synchronization messages (SynMsg) exchanged among the communicating SOs by the radio section.

The radio section is in charge of receive SynMsgs from communicating SOs and to send, in broadcast modality, the SynMsg each $t_{k}=kT_{P},k\in N^{+}$, where *T_P_* is the synchronization time period. The message SynMsg_ji_, sent at $t_{k}$ by SO#j and received by SO#i, includes: $\left\{id_{j},{\hat{\alpha }}_{j}\left(t_{k}\right),{\hat{o}}_{j}\left(t_{k}\right),\tau_{j}\left(t_{k}\right)\right\}$, where $id_{j}$ is the SO identifier that is unique for each SO.

**Figure 1:**
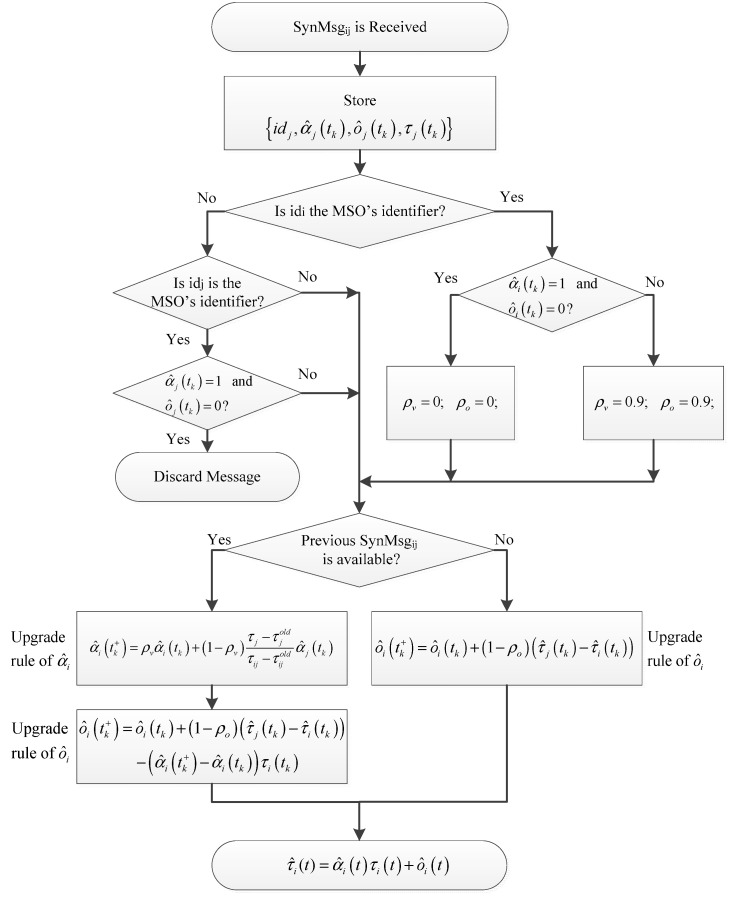
Block scheme of the synchronization procedure executed by the *i*-th object (SO or MSO), when receiving a SynMsg_ij_ from the *j*-th object.

At the reception of the SynMsg_ji_, the radio section communicates this event to the μ-processor that starts the synchronization procedure of the block scheme of Figure 1.

In Figure 1, $\tau_{ij}$ is the number of clock impulse of SO#i at the reception of SynMsg_ij_. The variables $\tau_{j}^{old}$ and $\tau_{ij}^{old}$ refer to the previous reception of SynMsg_ij_, $\rho_{v}$ and $\rho_{o}$ are design parameters (memory factors) that can be set in the range [0, 1], and $t_{k}^{+}$ indicates the upgraded value of the time dependent variables ${\hat{\alpha }}_{i}\left(t\right)$, and ${\hat{o}}_{i}\left(t\right)$, respectively [31]. In particular, if $\rho_{v}$ = 0 and $\rho_{o}$ = 0, the node will correct its ${\hat{\alpha }}_{i}\left(t\right)$ and ${\hat{o}}_{i}\left(t\right)$ on the basis of the time values of its neighbors only, and vice-versa: if $\rho_{v}$ = 0.9 and $\rho_{o}$ = 0.9, the upgrade of ${\hat{\alpha }}_{i}\left(t\right)$ and ${\hat{o}}_{i}\left(t\right)$ will mainly depend on its own value.

The MSO is used to bring the sense of the time from one SO to another. If it executes the synchronization procedure for the first time, the SOs ignore its message. This condition is checked by the ${\hat{\alpha }}_{j}\left(t\right)$ and ${\hat{o}}_{j}\left(t\right)$ values in the message received from the MSO and verified that they are equal to the initialization values.

In order to speed up the convergence of the MSO to the common sense of time of the network and to avoid that it could perturb the network synchronization, at the first synchronization time instant (*t_k_*, with *k* = 1), the MSO takes the sense of time of the first received message. This condition is checked by the MSO by its own ${\hat{\alpha }}_{i}\left(t\right)$ and ${\hat{o}}_{i}\left(t\right)$ values and verified that they are equal to the initialization values. At the end of the first synchronization time instant (*t_k_*^+^, with *k* = 1), MSO will have ${\hat{\tau }}_{i}\left(t\right)$ bounded by the maximum and minimum values of the ${\hat{\tau }}_{j}\left(t\right)$ of the SOs in its BR, because it: (i) is in the same neighborhood of the visited SO, (ii) receives the same synchronization messages, and (iii) executes the same upgrade of ${\hat{\alpha }}_{i}\left(t\right)$ and ${\hat{o}}_{i}\left(t\right)$. In the successive synchronization time instants (*t_k_*, with *k* > 1), MSO will have ${\hat{\tau }}_{i}\left(t\right)$ bounded by the maximum and minimum values of the ${\hat{\tau }}_{j}\left(t\right)$ of the SOs.

The number of connections in the paths among SOs influences the time with whom the information of frequency and offset of each SO clock is shared among all SOs. In particular, the fully connected topology represents the best case, because each SO is directly connected to all others. The worst case is if a nonconnected SO is present. The isolated SO cannot be synchronized, because no information is shared with it.

Because the MSO moves, it increases the number of paths among SOs, creating new connections, but these connections vary in time according with the MSO route.

In the following Section 3, the conditions making the synchronization procedure success are investigated, and the effect of the MSO route on *σ* and ∆*_TIS_* is analyzed.

## 3. Effect of MSO on the IoT Network Synchronization

### 3.1. Convergence Assessment

An effect that must be taken into account by introducing the MSO in an IoT wireless network synchronized by the consensus-based technique of Section 2 concerns the convergence assessment to a common sense of time. The MSO spreads its sense of time in the network and potentially can make the consensus process fail [26]. A formal proof that the synchronization procedure of the Section 2 makes the consensus process successful is to verify the convergence condition of the Theorem#1 of [26]. Theorem#1 assesses that the consensus is stable, i.e., the SOs converge to a common sense of time, if the variable estimated by MSO is always bounded by the maximum and minimum estimation of the corresponding variables of each SO. At the starting of the synchronization procedure, the SOs ignore the MSO’s message, and the MSO takes the sense of time of the first received message. At the successive synchronization steps, the MSO and the SOs will execute the same synchronization procedure. Therefore, the MSO’s ${\hat{\tau }}_{i}\left(t\right)$ is always bounded in the maximum and minimum values of the SOs, satisfying the Theorem#1.

### 3.2. Reduction of σ Value

Another effect, important for measurement purposes, is the reduction of the synchronization accuracy *σ*. This effect is not considered in [26] and in the theoretical approaches, since they assume that, after the convergence, the difference between the estimated time values of any couple of SOs is zero. This condition cannot be achieved in practical cases, due to the nonidealities of the SO’s hardware components. In [31], the effect of the nonidealities is modeled by introducing the bounded time-varying disturbance vector *d(t)*, with maximum value *d_max_*, that depends on the hardware characteristics of the SO clocks as the clock noise, the quantization error introduced by the limited resolution of the clocks, and so on. The clock hardware characteristics determine the lower bound of the achievable synchronization accuracy. The SOs and the MSO update the hardware clock value each *T* seconds, according to (1) at *t_s_*_+1_ *= t_s_ + T*:

(3) $$\tau_{i}\left(t_{s+1}\right)=\tau_{i}\left(t_{s}\right)+\bar{\alpha }T+d_{i}\left(t_{s}\right),$$

where *s* is positive integer, $\bar{\alpha }$ is the clock frequency nominal value, and $d_{i}\left(t_{s}\right)$ is the i-th element of *d*(*t*) such that $\left\|d_{i}\left(t_{s}\right)\right\|\leq d_{\max}$. As a consequence, at *t_s_*_+*m*_ *=* (*s + m*)*T*, *m* positive integer (without executing the synchronization procedure) it is:

(4) $$\tau_{i}\left(t_{s+m}\right)=\tau_{i}\left(t_{s}\right)+m\bar{\alpha }T+d_{i}\left(t_{s}\right),$$

where $\left\|d_{i}\left(t_{s}\right)\right\|<d_{\max}$.

**Figure 2:**
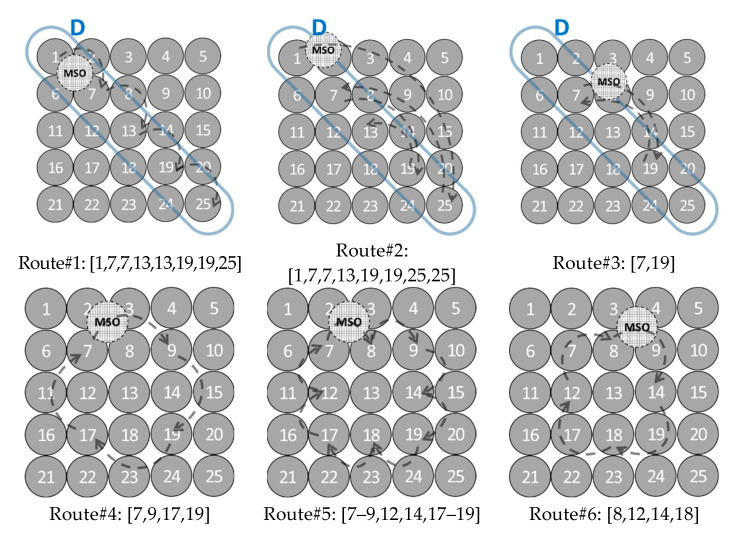
Different routes for N = 25 lattice topology.

At *t_s_ = t_k_* and *m* = *m_synch_ = T_P_*/*T*, the synchronization time occurs. By considering, for sake of simplicity, the case where only offset synchronization is performed, it is assumed that ${\hat{\alpha }}_{i}\left(t_{k}^{+}\right)-{\hat{\alpha }}_{i}\left(t_{k}\right)=0$. According to (2) and the upgrade rule of ${\hat{o}}_{i}$, Figure 2, it is:

(5) $${\hat{\tau }}_{i}\left(t_{k}^{+}\right)=\tau_{i}\left(t_{k}\right)+{\hat{o}}_{i}\left(t_{k}\right)+\left(1-\rho_{0}\right)\left({\hat{\tau }}_{j}\left(t_{k}\right)-{\hat{\tau }}_{i}\left(t_{k}\right)\right)={\hat{\tau }}_{i}\left(t_{k}^{}\right)+\left(1-\rho_{0}\right)\left({\hat{\tau }}_{j}\left(t_{k}\right)-{\hat{\tau }}_{i}\left(t_{k}\right)\right),$$

By considering a synchronous communication model among SOs and MSO, from (5), it is possible to evaluate the update of the software clock time of each SO and of the MSO. It is

(6) $$\hat{\tau }\left(t_{k}^{+}\right)=\left(I+\varepsilon L\right)\hat{\tau }\left(t_{k}\right),$$

where $\hat{\tau }\left(t_{k}^{+}\right)={\left[{\hat{\tau }}_{1}\left(t_{k}^{+}\right),\ldots ,{\hat{\tau }}_{Nc+1}\left(t_{k}^{+}\right)\right]}^{T}$**, $\hat{\tau }\left(t_{k}\right)={\left[{\hat{\tau }}_{1}\left(t_{k}\right),\ldots ,{\hat{\tau }}_{Nc+1}\left(t_{k}\right)\right]}^{T}$, *I* is identity matrix (*Nc* + 1) × (*Nc* + 1), *L* is the Laplacian matrix depending on the connections of the communication graph including the MSO, and $\varepsilon =1-\rho_{0}$. In Equation (6), ${\hat{\tau }}_{i}\left(t_{k}^{+}\right)$ is the sum of all the contributes, evaluated according to Equation (5), of the software clocks of the SOs and MSO in the BR.

From Equation (4), because no communication is performed between two consecutive synchronization time instants *t_k_* and *t*_*k*+1_, the software clock values are:

(7) $$\hat{\tau }\left(t_{k+1}\right)=\hat{\tau }\left(t_{k}^{+}\right)+1_{\left(N_{c}+1\right)\times 1}\bar{\alpha }T_{P}+d\left(t_{k}\right),$$

where $d\left(t_{k}\right)={\left[d_{1}\left(t_{k}\right),\ldots ,d_{\left(Nc+1\right)}\left(t_{k}\right)\right]}^{T}$. It is bounded as ${\left\|d\left(t_{k}\right)\right\|}_{\infty }<d_{\max}$. By Equation (6), Equation (7) becomes:

(8) $$\hat{\tau }\left(t_{k+1}\right)=\left(I+\varepsilon L\right)\hat{\tau }\left(t_{k}\right)+1_{\left(N_{c}+1\right)\times 1}\bar{\alpha }T_{P}+d\left(t_{k}\right),$$

According to graph theory [32,33], if the network graph is connected, the matrix I+εL has only one eigenvalue with value equal to 1, $\lambda_{1}=1$, while all the others are strictly lower than one (${\left|\lambda \right|}_{i}<1,i=2,\ldots ,\left(Nc+1\right)$). In addition, the eigenvector associated to $\lambda_{1}$ is the unit vector $v_{1}$ with dimension *Nc* + 1.

To evaluate the influence of the MSO on the synchronization accuracy, it is defined the synchronization delay between SO#*i* and SO#*j* as $y_{ij}\left(k\right)=C_{ij}\tau \left(t_{k}\right)$, where $C_{ij}={\left(e_{i}-e_{j}\right)}^{T}$ and $e_{i}$**, $e_{j}$ are the *i*-th and *j-*th vectors of the canonical basis. Since $C_{ij}v_{1}=0$, i.e., an object is with delay zero respect to itself, the $v_{1}$ does not contribute to the synchronization delay, and, consequently, on the synchronization accuracy. Moreover, the eigenvalues $\lambda_{1}$ has no influence on the synchronization delay. Since all the other eigenvalues are strictly lower than 1, the maximum value of the synchronization delay is bounded with respect to *d_max_*, as follows:

(9) $${\left\|y_{ij}\left(k\right)\right\|}_{\infty }<l_{1}d_{\max}\;\forall k,$$

where $l_{1}=\sum_{r=1}^{Nc}\sum_{k=0}^{\infty }\left|C_{i,j}{\left(I+\varepsilon L\right)}^{k}e_{r}\right|$ is the 1-norm of the impulsive response matrix between the disturbance vector and the output $y_{ij}$ [33].

Equation (9) shows that the maximum value of the synchronization delays, and consequently, the synchronization accuracy is linked in a proportional way with the parameter *l*_1_ that is influenced by the second largest eigenvalue of the matrix, which depends upon the connectivity of the graph. The more the graph is connected, the lower is the *l*_1_ value and, therefore, the influence of the disturbances on the synchronization accuracy. The MSO, by communicating with the SOs in its BR, creates new connections and, consequently, new path among SOs, increasing the connectivity of the graph.

### 3.3. Reduction of *∆*_TIS_ Value

Further effect of MSO concerns with the reduction of ∆*_TIS_*. Indeed, the lower is ${\left\|y_{ij}\left(k\right)\right\|}_{\infty }$, the earlier the synchronization delay between any couple of SOs becomes lower of the admissible delay range. Consequently, more the graph is connected, the lowest is the ∆*_TIS_* value. This result is also confirmed by Theorem#3 of [26], assessing that, if the MSO is used, the ∆*_TIS_* is reduced with respect to the case of a static network, i.e., only SOs. This theorem is based on the comparison of the second eigenvalue of the matrix I+εL in the two-abovementioned cases. In particular, it is proved that, with the MSO, the second eigenvalue of the I+εL is higher with respect to the case the MSO is not used, then the second eigenvector is lower, i.e., the network converges faster.

Nevertheless, the quantitative analysis of the ∆*_TIS_* reduction is still an open challenge [26].

## 4. Evaluation of the Effect of MSO on the IoT Network Synchronization

In order to evaluate the effect of the MSO on the synchronization, it is necessary to evaluate not only the increasing number of connections due to the newly introduced paths but also how such connections are spatially distributed. Indeed, in the extreme case that a SO is not linked to the network, if it is periodically visited by the MSO, as in [34], the synchronization of the whole network is achieved. If the MSO visits only the connected SOs, it is still not possible to achieve the synchronization of all the SOs.

To evaluate the effect of the MSO route on the network connections, a lattice network topology is considered. In this topology, all the SOs (except the ones on the boundary) have the same number of connections. In this case, the MSO effect on the connections is evaluated by two statistical parameters: the mean and standard deviation of the minimum number of connections among any couple of nodes on the observing time window. In the following, node refers to both SOs and MSO. In particular, because the nodes exchange the SynMsg each *T_P_*, the effect of the MSO on the connections among nodes is evaluated at each synchronization time *t_k_*. By considering at *t_k_* the network connection’s adjacency matrix *A*(*t_k_*), with dimension (*Nc* + 1) × (*Nc* + 1), the average minimum number of connection among nodes *μ_con_*, activated from the start of the synchronization procedure up to *t_k_*, is:

(10) $$\mu_{con}=mean\left(DST\left(\sum_{i=1}^{k}A\left(t_{k}\right)\right)\right),$$

The standard deviation is:

(11) $$\sigma_{con}\left(t_{k}\right)=st.dev.\left(DST\left(\sum_{i=1}^{k}A\left(t_{k}\right)\right)\right),$$

The element $a_{ij}\left(t_{k}\right)$ of the matrix $A\left(t_{k}\right)$ is the number of connections between node#i and node#j at $t_{k}$ as a consequence, the element in position *i*,*j* of the matrix $\sum_{i=1}^{k}A\left(t_{k}\right)$ is the number of connections between node#i and node#j available from the start of the synchronization procedure up to $t_{k}$.

The operator *DST*( ) of Equations (10) and (11) receives as input the adjacency matrix and returns the matrix where the element dst_ij_ is the minimum distance between node#i and node#j. The operator *mean( )* of Equation (10) returns the mean value of all the element of the matrix. The operator *st.dev.*( ) of Equation (11) returns the standard deviation of all the element of the matrix.

Because the synchronization parameters ∆*_TIS_* and *σ* depend on (i) the network topology, (ii) the number of SOs, and (iii) the MSO’s routes, numerical tests are designed to assess the theory in Section 3. In this way, the relationships among ∆*_TIS_*, *σ* $\mu_{con}\left(t_{k}\right)$, and $\sigma_{con}\left(t_{k}\right)$ [28] are highlighted. These relationships allow the establishment of the more convenient criteria in the design of the MSO route.

In the tests, the MSO visits the SOs in the predefined route. Since the MSO propagates the common sense of time at $t_{k}$, it estimates ${\hat{\tau }}_{i}^{}\left(t_{k}^{+}\right)$ according to the synchronization parameters of the last visited SOs (see Section 2). It is assumed that, at $t_{k}$, the MSO (dashed node in Figure 2) is directly connected with the visited SO and the SOs are directly connected to the visited SO. Both MSO and SOs communicate in wireless broadcasting mode.

In order to easily understand the effect of the MSO on the SO connections, a square lattice topology with different values of *Nc* is considered.

The *T_clk_* and ${\hat{\tau }}_{i}^{}\left(t_{0}\right)$ values are selected, respectively, according to the hardware realization of the clock typically equipping commercial MSO and SO [30] and the time required to power-on all the SOs and the MSO. In particular, *T_clk_* is selected equal to 1 ms with variation in the range [0.999980, 1.000020] *T_clk_*, ${\hat{\tau }}_{i}^{}\left(t_{0}\right)$ in the range [0, 30] × 10^7^ *T_clk_*. The observation time interval of each test is set equal to 10,000 s, and each node collects its own time interval each *T* = 2 *σ* and sends a SynMsg each *T_P_* = 10 s.

To evaluate the effect of the MSO, different MSO’s routes are considered. Among all these routes, in the following, particular interest is devoted to two different sets.

One set that includes straight-line routes, the main diagonal (Route#1–3 of Figure 2), is selected, because it splits symmetrically the network. The selection of the second diagonal or the central row or central column would not give further information, since they are a rotation of the main diagonal respect to the center of the matrix. Therefore, the results referred to these routes are not reported. Another set includes the routes selected with the following two criteria (Route#4–6 of Figure 2): (i) peripheral nodes of the network are visited, and (ii) the number of the node connections at each *t_k_* is maximized.

Random topologies of the network are not considered in this research, because they make difficult to understand the relationship between the MSO’s route and ∆*_TIS_*, since ∆*_TIS_* depends not only on the number of the visited nodes but also on their position in the network and the order with which MSO visits the nodes.

Let *N_i,j_*, *i*, *j* = 1, …, n, the node of the square lattice topology, where *n = sqrt*(*Nc*). Imposing that at time *t_k_*, MSO is on *N_i,j_*, the following routes are selected:

- Route#1:
  $\{\begin{array}{c} i=j=k\mathrm{mod}n\\ i=j=n \end{array}\begin{array}{c} k\mathrm{mod}n\neq 0\\ k\mathrm{mod}n=0 \end{array},\;k\in N^{+}$
- Route#2:
  $\{\begin{array}{cc} i=j=V[p] & p=k\mathrm{mod}length\left(V\right)\\ V=\left[1;n;2;n-1;\ldots ;\left\lfloor \frac{n}{2}\right\rfloor ;\left\lceil \frac{n}{2}\right\rceil +1;\left\lceil \frac{n}{2}\right\rceil ;\left\lceil \frac{n}{2}\right\rceil +1;\left\lfloor \frac{n}{2}\right\rfloor ;\ldots ;n-1;2;n\right] & \end{array},\;k\in N^{+}$
- Route#3:
  $\{\begin{array}{cc} i=j=V[p] & p=k\mathrm{mod}length\left(V\right)\\ V=\left[2;n-1;\ldots ;\left\lfloor \frac{n}{2}\right\rfloor -2;\left\lfloor \frac{n}{2}\right\rfloor +2;\left\lfloor \frac{n}{2}\right\rfloor ;\left\lfloor \frac{n}{2}\right\rfloor +2;\left\lfloor \frac{n}{2}\right\rfloor -2\ldots ;n-1\right] & \left\lceil \frac{n}{2}\right\rceil \mathrm{mod}\left(2\right)=0\\ V=\left[2;n-1;\ldots ;\left\lfloor \frac{n}{2}\right\rfloor ;\left\lceil \frac{n}{2}\right\rceil ;\left\lfloor \frac{n}{2}\right\rfloor ;\ldots ;n-1\right] & \left\lceil \frac{n}{2}\right\rceil \mathrm{mod}\left(2\right)\neq 0 \end{array},\;k\in N^{+}$
- Route#4:
  $\{\begin{array}{cccc} i=2, & j=2 & & k\mathrm{mod}\left(4\right)=1\\ i=2, & j=n-1 & & k\mathrm{mod}\left(4\right)=2\\ i=n-1, & j=n-1 & & k\mathrm{mod}\left(4\right)=3\\ i=n-1, & j=2 & & k\mathrm{mod}\left(4\right)=0 \end{array},\;k\in N^{+}$
- Route#5:
  $\{\begin{array}{cccc} i=2, & j=2 & & k\mathrm{mod}\left(8\right)=1\\ i=2, & j=\frac{n}{2} & & k\mathrm{mod}\left(8\right)=2\\ i=2, & j=n-1 & & k\mathrm{mod}\left(8\right)=3\\ i=\frac{n}{2}, & j=n-1 & & k\mathrm{mod}\left(8\right)=4\\ i=n-1, & j=n-1 & & k\mathrm{mod}\left(8\right)=5\\ i=n-1, & j=\frac{n}{2} & & k\mathrm{mod}\left(8\right)=6\\ i=n-1, & j=2 & & k\mathrm{mod}\left(8\right)=7\\ i=\frac{n}{2}, & j=2 & & k\mathrm{mod}\left(8\right)=0 \end{array},\;k\in N^{+}$
- Route#6:
  $\{\begin{array}{cccc} i=2, & j=\frac{n}{2} & & k\mathrm{mod}\left(4\right)=1\\ i=\frac{n}{2}, & j=n-1 & & k\mathrm{mod}\left(4\right)=2\\ i=n-1, & j=\frac{n}{2} & & k\mathrm{mod}\left(4\right)=3\\ i=\frac{n}{2}, & j=n-1 & & k\mathrm{mod}\left(4\right)=0 \end{array},\;k\in N^{+}$

Figure 2 shows the routes in the case *Nc* = 25 and the SOs visited by MSO. The dashed arches are the temporary connections created by the MSO in the route.

To evaluate ∆*_TIS_*, the admissible delay range equal to 100 *T_clk_*_,_ is imposed; *σ* is evaluated on the last 1000 s of the observation time interval. This time interval is selected on the basis of preliminary tests to guarantee that *σ* is evaluated when the common sense of time among the SOs is achieved.

**Figure 3:**
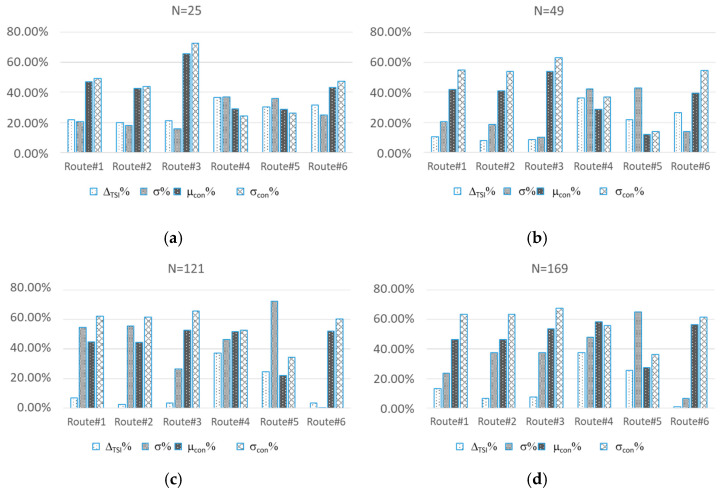
Percentage improvement of ∆*_TIS_* and *σ* and percentage variation of *μ_con%_* and *σ_con%_*, with respect to the case of static topology.

Figure 3 shows the percentage improvement of ∆*_TIS_* and *σ* and percentage variation of *μ_con_* and *σ_con_*, with respect to the case of static topology.

The numerical results show that, in all cases, the MSO increases the number of connections among SOs. Lower *σ_con_%* corresponds with higher improvement in *σ*. Concerning ∆*_TIS_*, according to the theory, the MSO improves its values, but the quantitative analysis is still an open challenge, and it will be studied in future research.

**Figure 4:**
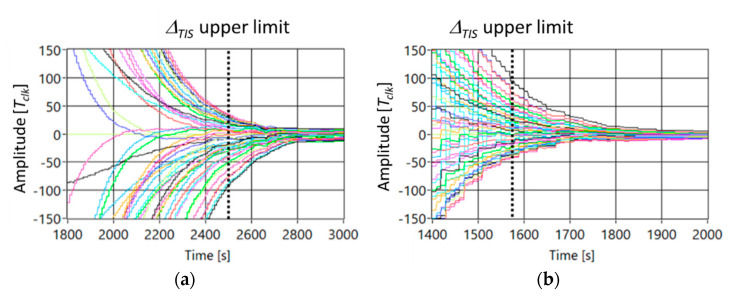
Trend of the delay among nodes in the case *Nc* = 49 and (**a**) static topology, and (**b**) MSO is moving according to route#4.

Figure 4 shows the trend of the delay between node#25 taken as a reference and all the others in the case Figure 4a static topology and Figure 4b MSO moving according to route#4.

By analyzing the numerical data, ∆*_TIS_* and *σ* are reduced of the 35% and 36%, respectively.

**Figure 5:**
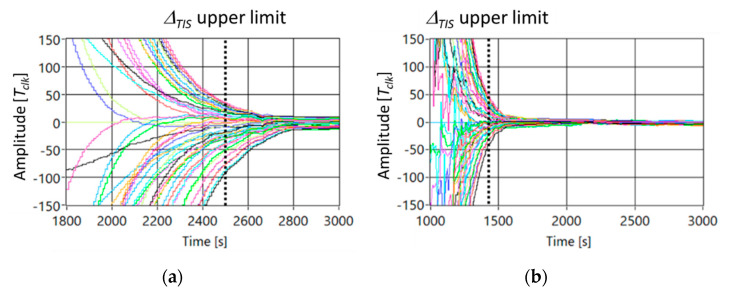
Trend of the delay among nodes in the case *Nc* = 49 and (**a**) static topology, and (**b**) MSO is randomly moving.

For sake of completeness, a random movement of the MSO is considered, and Figure 5 shows the trend of the delay among nodes in the case *Nc* = 49 and (a) static topology, and (b) MSO is randomly moving. In particular, the position of the MSO is established, time by time, by extracting an integer number in the range 1–49. As shown in Figure 5, as foreseen from theory, this random path gives better results with respect the other paths. In particular, ∆*_TIS_* is equal to 1400 s, and *σ* is equal to 10 *Tclk*. Such result is justified by the fact that the probability that the MSO links far nodes is increased.

## 5. Experimental Tests and Discussion

Experimental tests were carried out to evaluate the effect of MSO in a real scenario. 

**Figure 6:**
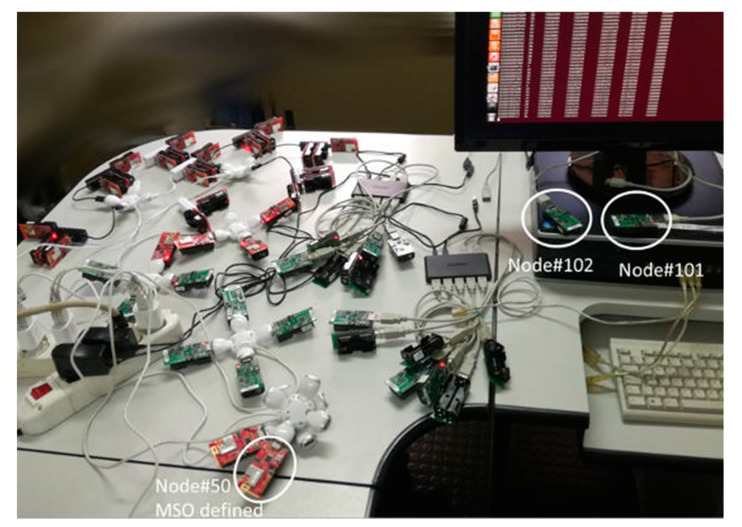
Experimental testbed.

The experimental testbed consisted of 23 Crossbow TelosB nodes, 30 xm1000 nodes and a PC. In particular, 21 TelosB nodes (indexed from 1 to 21) and all the xm1000 nodes were used to implement the synchronization algorithm. The TelosB node (indexed as 101) was plugged into the PC and used as ZigBee/USB protocol converter. The MSO is implemented in a xm1000 node indexed as 50. The TelosB node (indexed as 102) was used to trigger the 50 nodes to send the service messages containing their identification number *id_i_*, the software clock frequency ${\hat{\alpha }}_{i}\left(t\right)$, the software clock offset ${\hat{o}}_{i}\left(t\right)$, and the hardware clock *τ_i_* value to the ZigBee/USB protocol converter (and, in turn, to the PC) at sampling period *T* = 2 s. The data collected by the PC was used to evaluate the synchronization accuracy in terms of discrepancy among the software clocks ${\hat{\tau }}_{i}(t)$ for all *i* ∈ [1, 50]. Figure 6 shows the actual realization of the testbed.

In the TelosB [32] and xm1000 [35], the microcontroller is a TIMSP430 featuring a Digitally Controlled Oscillator (DCO) running at 8 MHz with a clock period *T_DCO_* = 0.125 μs and 10 kbyte of RAM.

Furthermore, it also features an External Crystal Oscillator (ECO) running at 32,768 Hz. Regarding the radio equipment, the nodes feature the IEEE 802.15.4/ZigBee-compliant module equipped with a RF transceiver operating within the range 2.4000–2.4835 GHz, compatible with ISM band, which allows a data rate of 250 kbps. An important feature of the radio chip CC2420 is the MAC-layer timestamp capability. This allows each node to read the local clock at the beginning of the transmission or reception of the start frame delimiter (SFD) of a message. This mechanism strongly reduces the random delays introduced by the transmission and the readings of the synchronization messages. Indeed, this mechanism was used in [36] to support their major assumption, i.e., communication delays are negligible with respect to *T_clk_*. The adoption of the precision tag “TMilli” giving a millisecond resolution of *T_clk_* was not cosmetic, as there are several hardware platforms, such as the Memsic’s IRIS mote [37], for which this represents the only option available. Thus, this reflects a realistic operating condition in several application contexts. 

TelosB and xm1000 motes have been programmed by TinyOS, an open-source operating system specifically designed for WSN [38]. 

In order to allow SOs and MSO to periodically send their clock time values to Node#101 and then to the PC, all them must be in the Node#101 BR [28,31,36]. To this aim, the nodes were deployed in a region of approximately one square meter. The high spatial density of nodes was chosen to evaluate the effectiveness of the proposed algorithm in a saturated spectrum network. For each node *i*-th, the neighborhood was predefined in order to achieve a lattice topology. 

As a consequence, the lattice topology and the movement of the MSO are realized by defining in each SOs and in the MSO a time variant Boolean vector *ND_i_*(*t_k_*) of the neighbors with *i* = 1…(*Nc* + 1). If *ND_i_*(*t_k_*)[*j*] = 1, the *j*-th node, at time *t_k_*, is in the neighborhood of the i-th node. The MSO routes are implemented by changing the update rules of *ND_i_*(*t_k_*).

The following parameters were used: *ρ_v_* = 0.9 and *ρ_o_* = 0.9. These are memory factors [36], and then their values influence the speed with which the whole network converges to a common sense of time. In order to compare numerical and experimental tests, *ρ_v_* and *ρ_o_* are the same for all the tests.

**Figure 7:**
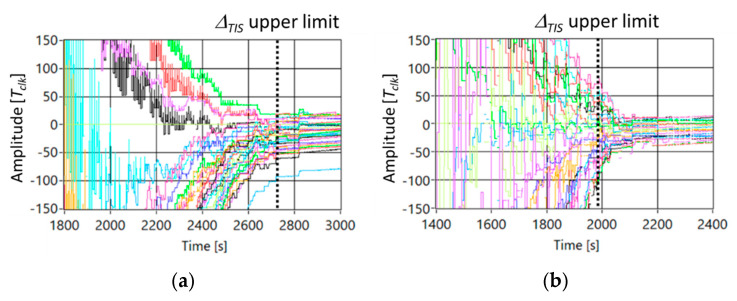
Experimental results of the delay among nodes in the case Nc = 49 and, (**a**) static topology, and (**b**) MSO moves according to the route#4.

Figure 7 shows the trend of the delay between SO#25 as reference and all other nodes of the network in the case (a) static topology, and (b) MSO moves according to the route#4.

By comparing Figure 7 with Figure 4, the same trend of the delay among SOs can be noted. The reduction of the delay among nodes shows the robustness of the consensus to the packet loss, as proved in [28,36]. 

By analyzing the experimental data, ∆*_TIS_* and *σ* are reduced of the 28% and 38%, respectively.

## 6. Conclusions

To overcome the convergence challenge to a common sense of time in the case of a Mobile Smart Object (MSO), in the paper, a novel synchronization algorithm based on consensus is proposed. This challenge is introduced by the fact that an MSO makes the network topology time varying, provoking the continuous evaluation of the logical tree to spread the sense of time. The consensus does not need logical tree, and, in this paper, the proof of the convergence was given for the proposed algorithm. Theoretical results, here presented, have highlighted that the MSO boosts the time synchronization of the network. This result is logically justified by the fact that the MSO brings the common sense of time among SOs by increasing their connections according to its path. Τhe effects of the MSO’s route on the statistical parameters describing the number of connections during the time and then on the values of *σ* and the ∆*_TIS_* is also investigated both by numerical and experimental tests that give compatible results and confirm the theoretical findings. Consequently, the criteria to select the route reducing *σ* and the Δ*_TIS_* are given. Ongoing activities are concerned with the investigation of the effect of the distance among nodes, so as of the speed of the MSO in reaching SOs, on the ameliorant of the *σ* and the ∆*_TIS_* values. Moreover, the case of multiple MSOs cooperating in bring the common sense of the time to the SOs will be also taken into account.

## Data Availability

Not applicable.
